# Medical Society of the County of Erie

**Published:** 1904-02

**Authors:** Franklin C. Gram


### SOCIETY PROCEEDINGS.

### Medical Society of the County of Erie.

Reported by FRANKLIN C. GRAM, M. D., Secretary.

THE eighty-third annual meeting of the Medical Society of the County of Erie was held in the rooms of the Society of Natural Science, Buffalo, January 10, 1904, the president, Dr. Ernest Wende, taking the chair, at 10 o'clock a. m. The minutes of the semi-annual meeting, held June 9, 1903, were read and approved.

Resignations were received from Drs. E. H. Tweedy, Cora B. Lattin, Henry B. Lattin, and Marian Marsh. On motion of Dr. H. R. Hopkins these resignations were accepted and the respective parties notified that under the law they would still remain responsible to this society for their dues.

Dr. William Warren Potter, chairman of the Committee on Membership, presented the names of Dr. Bruce L. Cook, Dr. John Messmer, Dr. Marshall Clinton, Dr. Francis N. Pitass, and Dr. Regina Flood Keyes as applicants, and moved that they be elected members of the society. The motion was carried. The president, Dr. Ernest Wende, read his valedictory address. He emphasised the most important work accomplished by the society during the past year in the conviction of a notorious individual with a bad criminal record in several states, and enlarged the valuable work of the district attorney in this respect.

On motion of Dr. J. B. Coakley, a vote of thanks was tendered the retiring president for his impartiality and for his interesting address.

On motion of Dr. Wm. C. Krauss, a vote of thanks was tendered the district attorney, and particularly Mr. Frank Abbott, his first assistant, for securing the conviction referred to in the president's address.

The president appointed the following nominating committee: Drs. F. F. Hoyer, J. B. Coakley, H. Lapp, J. H. Grant, and R. S. Meyers.

The committee presented the following list of nominations: president, William C. Krauss; vice-president, John D. McPherson; secretary, Franklin C. Gram; treasurer, Edward Clark; librarian, William C. Callanan; chairman of committee on membership, William Warren Potter; board of censors. Drs. Henry R. Hopkins, John H. Grant, Henry Lapp, Bradley Dorr, Marshall Clinton.

These names were duly balloted for and the candidates declared elected.

Delegates to the Medical Society of the State of New York: Drs. Harry Mead, Edgar A. Forsythe, Benjamin F. Rogers, Joseph Burke, De Lancey Rochester, Charles G. Stockton, John Mesmer, L. G. Hanley, Carlos E. Bowman, Clarence Tyler, F. M. Gipple, Chauncey Pelton Smith, W. S. Renner, Bernard Bartow, P. H. Hourigan, Marshall Clinton, N. W. Wilson, W. C. Jolls, C. A. Clements, A. H. Briggs, E. E. Briggs, Walter D. Greene, Henry D. Ingraham, John B. Coakley, Frederick F. Hoyer, F. H. Milliner, F. E. Fronczak, Grover Wende, Wm. H. Thornton, Eugene A. Smith, Regina Flood Keyes, Henry Lapp, Thomas F. Dwyer, S. A. Dunham.

These names were balloted for and the candidates declared duly elected.

Dr. Matthew D. Mann read a paper entitled, Some points in the treatment of uterine displacements, which was discussed by Drs. William Warren Potter, H. E. Hayd, and DeLancey Rochester.

Dr. Edward Clark submitted his annual report as treasurer, which was referred to an auditing committee, consisting of Drs. C. A. Clements and E. E. Briggs, who reported the accounts correct. The treasurer's report showed that $316 were collected in dues; $47 were received as proceeds of a fine; $200 were paid the society's attorney, and after payment of state society dues and other incidentals, a balance of $15 was left in the treasury.

Dr. Henry R. Hopkins submitted the report for the board of censors, as follows:

Report Board of Censors, June 20, 1903-January 12, 1904.

*To Medical Society of the County of Erie:*

The undersigned as Board of Censors of your society, begs to submit the following report covering its work from the date of the last semi-annual meeting to the present time.

During this period a number of cases of alleged violations of the public health law have come to the attention of your board, all of which have been investigated by the board with the aid of its counsel. Eight of these cases were found to require action and all of these except two have now been disposed of. In one of the cases the person complained of died while investigation was in progress; in another the Board took action resulting in the offender leaving the city and state. In one of the cases the situation was so serious that the board decided upon a criminal prosecution of the offender and a warrant has been issued for his arrest.

The attitude of the board in all cases has been to prosecute only when the ends of justice would be promoted thereby; hence, it has been felt that in cases where persons have unwillingly and unintentionally violated the law the only action necessary was to take such steps as to effectually prevent the recurrence of violations. Consequently there has been but one public prosecution instituted by the board. This does not mean, however, that your board is averse to prosecuting vigorously and making a public example of serious offenders. In this connection we would state that in addition to the cases before referred to, all of which were of unusual importance, your board has discovered that a number of barbers in a certain section of the city have been regularly engaged in administering treatment known as wet-cupping and dry-cupping. The board has also discovered a number of midwives who have been in the habit of administering herbs, medicines and using instruments, and who have been regularly violating the laws of the state in these particulars. Upon investigation of all of these cases the board found that the violaters were acting without criminal intent. The practices have now been stopped, but it is the intention of the board in the future to prosecute without leniency in all cases which may hereafter arise, and to endeavor to have the violaters of the letter of the law severely punished.

The board begs to report further that the case of the People against A. J. Weichers is very nearly ready for argument before the appellate division. Weichers, it will be remembered, was convicted of conspiracy and was on March 31, 1903, sentenced to a term of nine months in Erie County penitentiary. A motion for new trial was made and denied and a motion for the granting of a certificate of reasonable doubt was also made and denied. Later, however, a second motion was made asking for the granting of a certificate of reasonable doubt and for a release of the prisoner on bail pending the decision of the appeal. This motion was based upon a legal technicality and upon it the hearing was granted, Weichers being released from the penitentiary, June 16, 1903, upon bail bonds aggregating $4,000.00, covering the case referred to and also the indictments pending. We are informed by the district attorney that the case will probably be argued on appeal within the next thirty days.

H. R. Hopkins,
F. E. Fronczak,
Henry Lapp.

The report was received and the board was authorised to continue its attorney, and was allowed $30 for detective expenses.

Dr. William Warren Potter moved to remit the dues of the secretary, treasurer, and librarian during their terms of service. The motion was adopted.

Dr. H. R. Hopkins called attention to the proposed legislation, by which the sick who are cared for by the city are to be sent to the County Hospital, instead of any hospital as heretofore. He moved that the committee on legislation of this society have authority to watch the progress of this bill and invoke the aid of the state society, if necessary, to prevent its passage.

Dr. DeLancey Rochester seconded the motion and spoke strongly against the bill, as did Dr. A. A. Hubbell and Dr. W. C. Callanan.

Dr. J. A. Pettit thought we ought to know what the bill really is before the society takes definite action.

Dr. Edward Clark explained some of the features of the bill, and said he thought Mayor Knight's idea was to give the poormaster power to send certain chronic patients, especially tubercular cases which are now scattered throughout hospital wards and infecting others, to the County Hospital.

The motion prevailed.

Memorial addresses upon members who died during the year 1903, were then delivered:

### IN MEMORIAM.

### HERMAN MYNTER, M. D.

Born in Karebeck, Denmark, September 2, 1845.
Died in Buffalo, N. Y., February 9, 1903.
Delivered before the Medical Society of the County of Erie at its eighty-third annual meeting upon request of the president,

By Henry Reed Hopkins, M. D.

Ninety-Seventh President of the Medical Society of the State of New York.

America suffers many wrongs in the matter of undesirable emigrants from army-ridden, poverty-stricken Europe. America also enjoys the precious privilege of being sought as the future home of many of Europe's sons, who are men of distinguished ancestry, of high attainments, of noble ideals, of unusual and remarkable preparedness for sharing in full measure in the boundless opportunities for self-command, self-development, self-knowledge, which constitute the priceless offering of America to humanity.

Our deceased fellow, in whose memory these lines are drawn, Doctor Mynter, was a conspicuous example and illustration of the power of free America to attract not the feeble, the oppressed and the helpless alone, but to win and hold the strong, the well-equipped, the reliant, the masterful of Europe's sons, and after this manner the old world does enrich and endow the new. In the free soil and the free air of America, it requires but few like Jacobi, Fenger, and Mynter to compensate for an entire shipload of hewers of wood and drawers of water.

Dr. Mynter's ancestors have been traced back ten generations with reliable data, and the name is found in various records back to 1360. The name Herman Münter occurs in 1375, when the death of a proconsul of that name is recorded. From this time the name Münter appears constantly in every century, in every case in a connection showing that the Münters were people of means and position. The home of the family till 1765 was Lübeck, Germany, formerly one of the free cities of the Hanseatic league. In the council chamber of Lübeck, now in use, are to be found the portraits of two first burgomasters, whose blood runs in the Münters veins. One is Herman Münter and the other Herman Kron, who represent the family in the generation of great, great grandfathers.

In the year 1765, Balthasar Münter, Dr. Mynter's great grandfather, was called to Denmark by King Frederick V., to be his spiritual adviser, German being then the court language of Denmark. This Balthasar Münter was a graduate of the University of Jena, having taken his master's degree there in 1757. He became the founder of the family in Denmark. His son, Dr. Frederich Christian Carl Heinrich Münter, was also born in Germany and was educated at the German universities of Gotha and Göttingen. He was an extensive traveler and orientalist and a patron of the arts and of artists, the Danish sculptor Thorwaldsen being one of the artists who was materially helped by his beneficence.

Dr. Frederich Münter attained greater eminence than any other member of the family; he became archbishop of Denmark, and it was while the episcopal palace in Copenhagen was his residence that his son Frederich, Dr. Mynter's father, was born. It is thus seen that Dr. Mynter's father was the first generation of the family to be born in Denmark. He was also the first to be educated there, taking his degree from the University of Copenhagen. He became pastor of the church of Karebeck (pronounced Carbeck), Denmark, and in this little village by the sea, his son Herman Mynter was born, September 2, 1845.

Herman received his education in the Latin school of Herlufsholm and the University of Copenhagen, where he was graduated in medicine, *cum honore,* in 1871. In 1872-73 he served in the Danish navy as surgeon, and in 1875 he came to America and to Buffalo, and began for the first time the independent practice of his profession. On his mother's side his ancestors were for generations members of the legal profession, as on his father's side they had been clergymen. His father intended him for the church, and with this in view he was started in a course of Hebrew, but the youth Herman, to use his own language, thought it good that, in a family where there had been so many preachers some one should practice, and so Hebrew was dropped and anatomy taken up. To his chosen profession he became absolutely devoted, and nothing was able to turn his thoughts from it except his love for historical reading. This was his only hobby. He believed that every physician should have a hobby to ride. One less in line with his work might have helped to prolong his life.

Dr. Mynter was a frequent contributor to the literature of his profession through medical and surgical periodicals, his subjects being for the most part surgical. In 1897 he published, both in English and Danish, a treatise on appendicitis. This was presented to his Alma Mater as his thesis for the degree of Doctor of Medicine. The thesis was accepted and the degree granted. A dispensation from the king, however, was needed to permit the granting of the degree in the absence of the recipient.

Dr. Mynter's love of his native country was one of his most cherished thoughts, and his enthusiasm for America was equally great. He was never known to utter one harsh, or unkind, or unjust criticism of America or Americans, and to be able to serve the country of his adoption gave him his keenest joy.

Dr. Mynter's success in his profession was immediate, conspicuous, and sustained. His preparation for his life work was unusual; as a physician he was conservative, wise and resourceful; as a surgeon he was profound, brilliant and heroic; as a teacher he was thorough, earnest and masterful.

From the many hundreds of letters of sympathy and condolence the sad event of his death brought from all parts of the world to his bereaved family, I gather the following as competent testimony of the real value of Dr. Mynter's life and character:

Rev. W. S. Hubbell, D. D., of New York and Plainfield, N. J.

I knew him (Dr. Mynter) pretty well before those days at the Sisters' Hospital, but during those weary weeks our attachment increased manyfold. We saw him day after day for nearly three months and saw him, too, at his best in the midst of his varied responsibilities and duties. Under his quick, peremptory manner, we found out his kindness of heart, his devotion to duty, his fierce hatred of all hypocrisy and humbug, his enormous capacity for work, his unselfish readiness to aid everyone in distress, his real gentleness with the suffering, and his conscientious resolve to leave nothing undone that could save any patient. We had many religious talks at my bedside and many amusing theological discussions. Perhaps he started these excursions into theology for the sake of diverting my mind, but often they sprang out of some fatal case in the wards near us, or some critical operation where he seemed to hold the life or death of his patient in his hand. But he used to agree that after all it was God's will that would be done, with his skill or in spite thereof. He was a devout man at the bottom of his heart, and lived with the fear of God before his eyes, despite a doctor's peculiar temptation to live in the natural realm alone.

Mr. W. H. Lecky of London, historian and member of parliament:

I saw much of Dr. Mynter at Nauheim last summer, and felt deeply his great charm, both intellectual and moral, and had always hoped that we might meet again. It then seemed as if Nauheim had done its healing work and I am deeply sorry to hear of his early death. It is a brilliant and most useful life cut short in its prime.

Constantine Brun, Minister of Denmark to the United States:

Denmark and every Dane has reason to be proud of Dr. Mynter, and I shall never forget the cordiality and genuine kindness with which he always greeted me when we met. It is a great disappointment to me that he should leave us so early.

Hon. James O. Putnam:

I, too, have in the death of Dr. Mynter lost a valued friend, and one of a noble manhood. His native country was proud of its distinguished son, and in his adopted country he won professional eminence and personal distinction. To this was added a happy home and friends who honored and loved him.

Mr. Herbert G. Lord, Columbia University:

Dr. Mynter was ever so fresh in feeling, so open and straightforward in word, so delightful to know. No one I ever knew was like him, with a characteristic touch all his own, saturating the very modulations of his voice as it did in turns of phrase and flash of eye. This made him stand out among one's friends as a marked and unusual man. I do not like to think that I shall never have converse with this man again. I cannot believe that such men are, with how long labor, formed to come up as the grass and be cut down. One must believe they are of too much worth to be lost, must keep their personal values somewhere for future joys given and received.

Dr. DeLancey Rochester:

It was my privilege to know Dr. Mynter well in the relation of disciple and master when I was interne at the General Hospital, and he was one of the attending surgeons. At that time I was impressed with the thoroughness of his education and with his good surgical judgment. His methods were an inspiration to the younger men to strive for similar attainment. His loss will be felt deeply by the medical and surgical world.

As years have advanced I have learned to know Dr. Mynter better and to appreciate highly both his medical knowledge and his social qualities. At any social gathering of men a pleasant time was assured if Mynter were there, and we will miss him greatly at such gatherings in the future.

Mr. L. G. Sellstedt:

I loved Mynter as I have loved few men. I doubt if any out of his own family better knew the noble and gentle spirit that dwelt in that body. His towering intellect was apparent to all who associated with him, as was his skill to his professional brethren, but the finer fibers of his soul were often hidden by that uncompromising honesty of character which could not prevent him from speaking what was in his mind.

Dr. H. Newton Hinneman of Bad Nauheim and Paris:

Dr. Mynter has left a high record behind him, an inheritance of which his family may justly be proud. He won the affection of the profession as well as that of a large clientèle, and as time goes on the name of Mynter will occupy a proud position in the records of the profession's great men. Surely there is consolation in this legacy, than which the millions in the banks pale into insignificance.

Headquarters, 65th Regiment, N. G. N. Y., Buffalo, N. Y.

The board of officers of the 65th Regiment at a regular meeting, May 14, 1903, adopted the following:

The members of the 65th Regiment desire to record their sorrow at the death of Herman Mynter, M. D., and extending their sympathy to his family in their affliction; also wish to express their appreciation of him as a citizen, and especially of the splendid service rendered by him to the sick of the 65th Regiment, N. Y. V. I., during the Spanish-American war. They desire to bear witness to the devotion and untiring effort which he gave on the hospital train by which the lives of as many as he could reach were spared.

*Resolved,* That a copy of this memorial be sent to the family of Doctor Mynter.

Samuel M. Welch, Colonel,
Albert H. Briggs, Major,
Charles E. P. Babcock, Major,
Henry W. Brendel, Captain.

I would emphasise two incidents in the life of Dr. Mynter as distinctly revealing the manner of man, those who knew him best knew him to be. Dr. Mynter was the first well known surgeon to reach the bedside of the dying President McKinley. As was his wont he was provided with suitable instruments for immediate use. His mind was also clear as to the only course of procedure for the case. Within a few moments he was joined by several of his colleagues; a brief consultation resulted in an agreement as to what should be done. The usages of his profession, which he knew well, gave him the opportunity and the right to announce in substance, "Very well, gentlemen, such being the case, I will ask you to prepare yourselves to assist me in making this operation, the only possible means of saving the life of the most powerful of the Rulers of Earth." Dr. Mynter was a general surgeon, having had a large experience in abdominal surgery. In the consultation was a surgeon of larger experience in abdominal surgery than Dr. Mynter, from the fact this was the other's special field, although generally considered a specialist in the diseases of women. To this surgeon, Dr. Mann, with a self-abnegation and courtesy seldom equaled and never surpassed, Dr. Mynter resigned the supreme opportunity of his life. Such a sublime act would have been impossible to any man save one entirely loyal to the highest ideals of our noble art. Few men ever have such a privilege; no man ever made better use of so supreme an opportunity.

It was my sad privilege to visit Dr. Mynter during the closing days of his final illness. Upon entering his chamber on one occasion I remarked, "How well you are looking this morning." His reply to my salutation was characteristic. With a graceful wave of the hand he said, "Of course. Why should I not look well. My good friends and wise physicians tell me I have not long to live. This morning I sent for my spiritual adviser. He left me but a few moments ago. If I am to die I will die as becomes a gentleman and a Christian."

In recalling these incidents I can but reflect that conduct and character after this manner comes only to those whose precious privilege it has been to drink from the sweet waters of life. In conclusion it seems not inappropriate to call to your attention the minutes of the special meeting of this society called to take action upon Dr. Mynter's death, at which the following was adopted:

Forasmuch as it hath pleased Almighty God, in His wise providence, to take out of the world the soul of our deceased brother, Dr. Herman Mynter, we, the Medical Society of the County of Erie, therefore declare and pronounce this estimate and testimonial of his life, work, and character.

Herman Mynter was generously endowed by an honorable ancestry, with rare gifts of mind and person. These gifts, fortunately, had been well developed by his educational training, the best the schools and universities of Europe could give; and when in the days of his early manhood he came to Buffalo to become a loyal and enthusiastic American citizen, and a member of its medical profession, he came not empty handed, but magnificently equipped; not needing care, but ready and able to care for others. During his entire life, as strength and health permitted, he practised his profession, which he ever honored and adorned, with devotion to its highest ideals, and with distinguished success.

Dr. Mynter was a frequent contributor to the literature of medicine, and his contributions ever bore the mark of distinct individuality, unusual mastery of its scientific principles and progress-making originality. His success as a teacher was pronounced, because he ever gave to this portion of his work,—as to all his efforts,—his very best. The monument of this life, more lasting and beautiful than carven granite, or sculptured marble, is his practice—where thousands of human lives have been blest by his intelligent conception of scientific principles and his skilful application of these principles for the relief of suffering, deformity, and disease.

We loved him and we will cherish his memory, not only for his brilliant professional achievements of mind and hand, but moreover for his record of clean, manly and blameless walk and conversation, the proof of the intrinsic nobility of his character. Therefore

*Resolved,* That this minute be accepted, inscribed upon our records, and a copy transmitted to the family of our deceased brother.

